# Determination of Azelastine in Human Plasma by Validated Liquid Chromatography Coupled to Tandom Mass Spectrometry (LC-ESI/MS/MS) for the Clinical Studies

**Published:** 2010-06

**Authors:** Yoo-Sin Park, Shin-Hee Kim, Young-Jae Kim, Seok-Chul Yang, Min-Ho Lee, Leslie M. Shaw, Ju-Seop Kang

**Affiliations:** 1 *The Institute of Biomedical Science, Hanyang University, Seoul, Republic of Korea;*; 2 *Department of Pharmacology, College of Medicine, Hanyang University, Seoul, Republic of Korea;*; 3 *Department of Obstetrics and Gynecology, College of Medicine, Hanyang University, Seoul, Republic of Korea;*; 4 *Department of Internal Medicine, Seoul National University Hospital, Seoul, Republic of Korea;*; 5 *Department of Internal Medicine, College of Medicine, Hanyang University, Seoul, Republic of Korea;*; 6 *Department of Pathology & Lab Medicine, College of Medicine, University of Pennsylvania, Philadelphia, PA 19104, USA;*; 7 *Department of Bioengineering, College of Engineering, Hanyang University, Seoul, Republic of Korea*

**Keywords:** azelastine, bioequivalence, LC-ESI/MS/MS, pharmacokinetics

## Abstract

A liquid chromatography coupled to tandem mass spectrometry (LC-ESI/MS/MS) was validated to determine azelastine in human plasma. Azelastine and internal standard (IS, clomipramine) were separated using a mobile phase of acetonitrile:(5 mM)-ammonium acetate solution (70:30, v/v, pH=6.4) with flow rate of 0.25 mL/min over YMC C8 column. One mL of plasma was extracted by n-hexane: 2-propanol (97:3, v/v) and then injected into HPLC system after reconstitution by acetonitrile: (5 mM)-ammonium acetate (1:1, v/v) solution. Detection was carried out on API5000 MS system by multiple reactions monitoring mode. The ionization was optimized using ESI (+) and selectivity was achieved at m/z 382.2→112.2 for azelastine and m/z 315.3→228.0 for IS. Total run-time (<2.0 min) and linearity (10 (LLOQ) ~5000 pg/mL) were good. No endogenous compounds were found around the retention time. The inter- and intra-day precision and accuracy were 4.13~17.91% and 87.57~109.70%, respectively. This validated method was successfully applied to a bioequivalence study in 23 healthy Korean male volunteers from the blood samples taken up to 96 h after orally administered 2 tablets of 1 mg of reference and test formulations of azelastine in a double-blind, randomized, cross-over design. The mean peak plasma concentrations (C_max_ ± SD) of 1.02 ± 0.37 and 1.10 ± 0.43 ng/mL were reached at 5.9 and 5.6 h for reference and test azelastine, respectively. The mean total area under the curve (AUC_0-infinity_) were 25.96 ± 10.84 and 28.24 ± 11.09 ng·h/mL for reference and test formulations, respectively. The reference and test azelastine formulations can be considered bioequivalent from the obtained pharmacokinetics by LC-ESI/MS/MS.

## INTRODUCTION

Azelastine hydrochloride is a novel chemical entity that has been developed as an long-acting anti-allergic and asthma-prophylactic agents that possesses properties with histamine-H1 receptor-antagonistic activity and antagonism of the various chemical mediators such as adenosine, leukotriene, endothelin-1, platelet activation factor (PAF) and superoxide-free radicals (1, 2). Azelastine prevents and attenuates chemical mediators- and exercise-induced bronchoconstriction (3, 4), therefore it has been used as asthma prophylactic and anti-allergic agents orally or nasally with long duration of action in adults and children (5, 6). The recommended oral dosage of azelastine for ‘prophylactic and maintenance therapy of bronchila asthma’ or ‘prophylaxis and treatment of allergic rhinitis’ in adults or children (>6 years)’ is 4 mg twice daily or 1~2 mg twice daily, respectively. Alternatively, for patients with nocturnal or early morning symptoms, single 8 mg oral dose may be recommend in the evening (7).

Azelastine is somewhat rapidly absorbed and gradually metabolized after oral administration, as showed that Tmax (4.2~5.19 h) and the elimination half-life (22.2~35.5 h), demonstrating high inter- and intra- individual variance according to the oral dosages (7, 8). Pharmacokinetic (PK) parameters of azelastine also showed different characteristics in young and elderly groups after single and multiple doses (9). Besides, azelastine administration of oral dosages has less side effects and higher tolerance than nasal dosage, resulted in broader use in the patients with different ages and disease symptoms (7, 8). Therefore, it is important that monitoring azelastine concentrations in the bloodstream at intervals after oral dosages in order to maintain a relatively constant concentration of azelastine. In this point, it is meaningful to investigate the pharmacokinectics of azelastine in different heath status, ages and races.

Plasma azelastine and its metabolites concentration measured by the analytical radioimmunoassay (RIA) and HPLC showed rough time courses in most of the subjects and pronounced inter- and intra-individual variations after single dose as well as after multiple doses (8, 9). Until now, azelastine and its several metabolites in biological matrix have been analyzed by RIA (7, 9), HPLC-fluorescence detection (8, 10), HPLC (11), HPLC mass spectrometry (MS) (12), electrokinetic capillary chromatography or HPLC-MS/MS (13). Most of these methods have long run-time, complicated procedures for sample preparation/analytic processes or low sensitivity with lower limits of quantification (LLOQ) of hundreds pg/mL, and these limitations have interfered with accurate pharmacokinetics of azelastine formulations.

A new sensitive LC-ESI/MS/MS method for azelastine detection was expected to have shorter run-time, simpler sample preparation and higher sensitivity of LLOQ than formal analytical methods. Therefore we validated and applied it in this double-blind, randomized, cross-over study to evaluate the bioequivalence (BE) by comparing PK parameters such as the area under the curve (AUC_0-t_), the AUC extrapolated to infinity (AUC_0-infinity_), the maximal concentration (C_max_), the time for maximal concentrations (T_max_), the plasma elimination half-life (T_1/2_) and the elimination constant (*Ke*) based on plasma azelastine concentration-time plots after an oral dose of 2 tablets of 1 mg, the most recommended dose/time (7), reference and test formulations of azelastine in 23 healthy Korean male volunteers.

## MATERIALS AND METHODS

### Chemicals and Reagents

**Figure 1:**
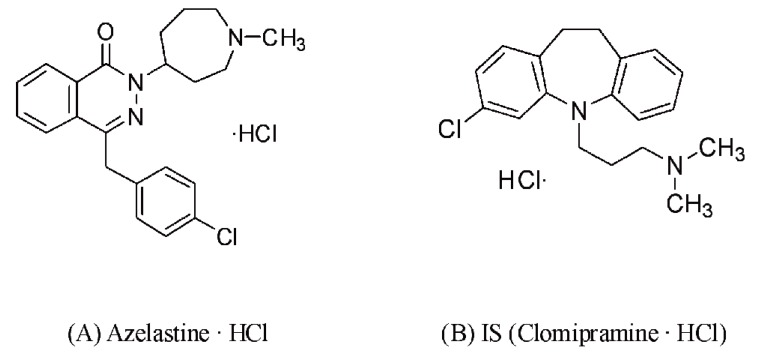
Chemical structures of azelastine·HCl (A) and IS, clomipramine·HCl (B).

Azelastine and internal standard (IS, clomipramine) as monohydrochloride compounds were obtained from DaitoThink Chemical LTD (Hangzhou, China) and Sigma Co. (St. Louis, MO, USA), respectively (Figure 1). HPLC grade acetonitrile, methanol and ammonium acetate were purchased from Sigma Co. (St. Louis, MO, USA). Other agents and solvents used were analytical grade, and water was purified by a Milli-Q system (Millipore Co. USA). Reference drug (Azeptin™, Bukwang Pharm. Co. South Korea) and test drug (Azelazen™, Newgen Pharm. Co. South Korea) containing 1 mg-azelastine·HCl per tablet was used in this study.

### Stock Solutions and Standards

Stock solutions of azelastine hydrochloride and IS were prepared with 50% methanol in distilled water solution to final concentrations of 1.0 mg/mL and 10 ug/mL, respectively. And then both stock solutions were stored at -20°C. A set of six non-zero calibration standards, ranging 10~5000 pg/mL (10, 50, 100, 500, 1000 and 5000 pg/mL) was prepared by spiking the blank drug-free human plasma with an appropriate amount of azelastine. The quality control (QC) samples at four concentration levels (10 (LLOQ), 30 (LOQ), 500 and 4000 pg/mL) were prepared in a similar manner to the calibration standards. Blank human plasma was tested before spiking to ensure that no endogenous interference was found at retention times of azelastine and IS.

### Preparation for Plasma Samples

A 1.0 mL aliquot of human plasma was spiked with 100 μL of standard solution of IS (10 μg/mL) and 5.0 mL of n-hexane-2-propanol (97:3, v/v) solution, and vortex-mixed for 10 min. After centrifugation at 4000 rpm for 5 min, clear aliquot was transferred to another tube, and was evaporated at 40°C using a nitrogen flow directed to the upper surface. The residue was reconstituted in 150 μL of acetonitrile:(5 mM)-ammonium acetate (1:1, v/v) solution by vortex mixing for 5.0 sec. 100 μL of this solution was transferred into an autosampler vial, and 7.0 μL was injected into the LC-ESI/MS/MS system.

### LC-ESI/MS/MS Conditions and Quantifications

The LC system used was NANOSPACE SI-2 3301 system (Shiseido Co., Japan) chromatograph equipped with an isocratic pump, NANOSPACE SI-2 3133 autosampler (Shiseido Co., Japan) and Peak Simple LC Data System with Analyst Software version 1.4.2 (Lab Alliance Co., State College, PA, USA). MS analysis was performed using an API 5000™ mass spectrometer system (Applied Biosystems / MDS SCIEX, Foster City, CA, USA) equipped with a turbo ion spray interface operating in the positive ion mode (ESI (+), 5500.0 V). This system was set to multiple reactions monitoring (MRM) mode which selects and dissociates parent ions to analyze the daughter selective ions with great selectivity and sensitivity.

The analytical column was YMC Pack Pro C8 column (2.0 × 50 mm, 3 μm; Waters Co. Milford, MA, USA). The mobile phase consisted of acetonitrile:(5 mM)-ammonium acetate (70:30, v/v). The flow rate was 0.25 mL/min and the injection volume was 7.0 μL. The dwell time was set at 5.0 sec, and the probe temperature was set at 400°C with ultra-high-purity nitrogen as curtain gas (30.0 L/min) and collision gas (5.0 L/min). A full-scan positive ion spectrum showed that the precursor ions were the protonated molecules, [M+H]+ of m/z 382.2 for azelastine and m/z 315.3 for IS. After collision-induced dissociation, the most abundant ion in the product ion mass spectrum was at m/z 382.2→112.2 for azelastine at a collision energy of 35.0 eV, and m/z 315.3→228.0 for IS at a collision energy of 53.0 eV. The strongest fragment of each compound was selected and used as Q3 ion to be monitored. Unit resolution was used for both Q1 and Q3 mass detection. The ion source parameters were set as follows; curtain gas=30.0 p.s.i., collision gas=5.0 p.s.i., nebulizer gas1=20.0 p.s.i., nebulizer gas2=16.0 p.s.i., ion spray voltage=5500.0 V, temperature=400°C. No significant interferences at the retention times of azelastine or IS were observed in the MS chromatography under the aforementioned LC-ESI/MS/MS conditions.

### Assay Validation

Calibration curves were based on peak area ratios of azelastine to IS for six calibration standards over the range of 10~5000 pg/mL for azelastine in human plasma analyzed in duplicate. Linearity was determined by linear least-squares regression with a weighting index of 1/x^2^ on the peak area ratios of azelastine/IS versus azelastine at the concentrations of the seven plasma standards (zero, 10 (LLOQ), 50, 100, 500, 1000 and 5000 pg/mL) to generate a calibration curve. Accuracy and precision were assayed from five replicates of QC samples on five different days at 10 (LLOQ), 30 (LOQ), 500 and 4000 pg/mL and analyzed by one-way ANOVA.

### Pharmacokinetics and Bioequivalence in Healthy Volunteers

We used most common statistical design for comparing two formulations that is a double-blind, randomized, standard 2 × 2 crossover design (14) to assess the bioequivalence between test and reference drugs of azelastine formulations. Twenty-three participants were informed the aims and risks of the study by the clinical investigator, and then written informed consents to participate in this study were obtained before enrollment. Participants had not taken medications (including over-the-counter) 2 weeks prior to or during the study period. The study was performed according to the revised Declaration of Helsinki (15) for biomedical research involving human subjects and the rules of Good Clinical Practice (GCP) (16). The Institutional Review Board (IRB) of Hanyang University Medical Center approved the protocol prior to the start of the study. Twenty-three volunteers aged 19~27 years (23.0 ± 2.1 years), with body weight 61.0~101.0 kg (72.1 ± 9.2 kg) and height 165.0~187.0 cm (175.9 ± 6.1 cm) were included in this study with a 1-week washout period. One of the volunteers withdrew from participation before completing this study. The participants were non-alcoholic and free from diseases, and were evaluated in physical examination and biochemical tests such as blood albumin, alkaline phosphatase, ALT, AST, glucose, creatinine, urea nitrogen, total cholesterol, protein, bilirubin, hemoglobin, hematocrit, total and differential white cell counts, and routine urinalysis. During each period, the volunteers were hospitalized to the clinical PK laboratory in Hanyang University Medical Center at 18:00 pm, and had an evening meal before 20:00 pm. After an overnight fasting, they received 2 tablets of 1 mg-azelastine formulation of test or reference drugs at 7:00 am along with 240 mL water. Subjects were in the seated position for at least 1 h and then fasted for 4 h. Standard lunch and evening meals were provided at 4 and 10 h after dosing. Liquid consumption was allowed ad libitum after lunch except xanthine and acidic beverages including tea, coffee and cola. At 0 and 1.5, 5, 12, 24, 48, 72 and 96 h after dose, blood pressure, heart rate and body temperature were recorded. Blood samples (8 mL) were withdrawn by indwelled catheter into heparin-containing tubes from a suitable antecubital vein before and at 0.5, 1, 2, 4, 6, 8, 10, 24, 48, 72 and 96 h after dose. The blood samples were centrifuged at 2500 rpm for 10 min at room temperature and plasma was stored at -70°C until analysis. The total plasma azelastine concentrations were determined as the mean of duplicate samples.

A noncompartmental PK method was employed to determine the PK parameters of azelastine. The maximal concentration (C_max_) and time for maximal concentrations (T_max_) were determined by visual inspection from each subject’s plasma concentration versus time plots for azelastine. PK analysis was performed by PK solutions software version 2.0 (17) and PKCALC computer program based on equation described by Shumaker (18). The area under the curve (AUC_0-t_) was calculated by the linear trapezoidal rule from 0 to 96 h. The AUC extrapolated to infinity (AUC_0-infinity_) was calculated as AUC_0-t_ + C_t_ / *Ke*, where C_t_ is the last measurable concentration, and the elimination constant (*Ke*) was obtained from the least square fitted terminal log-linear portion of the plasma concentration versus time profile (19, 20). Plasma elimination half-life (T_1/2_) was calculated as ln2/*Ke*. To assess the BE between the test and reference formulations, AUC_0-t_ and C_max_ were considered to be the primary variables, and 2-way analysis of variance (ANOVA) for the crossover–randomized design was used to assess the effect of formulations, period, sequence, and subjects on these parameters (17). Differences between 2 related parameters were considered statistically significant at p<0.05. The inclusion of 90% confidence intervals (CI) of the geometric mean for the individual test/reference ratios for AUC_0-t_ and C_max_ were obtained to assess the BE between formulations. The inclusion of the parametric 90% CI for the ratios in the 80~125% range proposed by the FDA was computed using parametric methods for log-transformed data (17, 20).

## RESULTS AND DISCUSSION

### Separation and Method Validation

**Figure 2:**
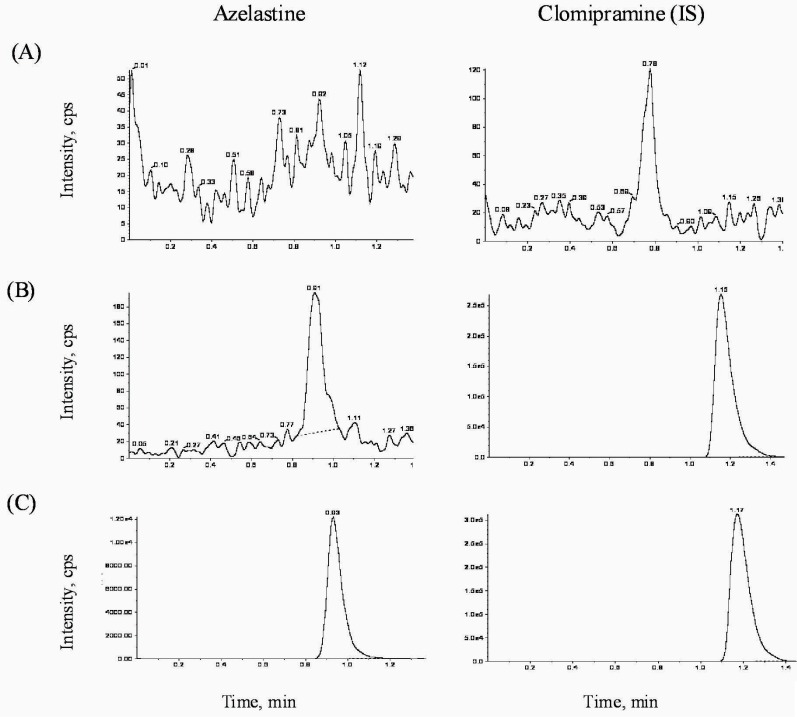
Ion chromatograms of (A) double blank plasma, (B) plasma with calibration standard of azelastine (10 pg/mL) and 100 μL of IS (10 μg/mL), and (C) subject’s plasma, which was spiked with 100 μL of IS (10 μg/mL), of 3 h after an oral administration of 2 tablets of 1 mg-azelastine.

The selective reaction monitoring chromatograms are presented in Figure 2; (A) double blank (no azelastine and no IS) human plasma, (B) plasma spiking with 10 pg/mL (LLOQ) of calibration standard of azelastine and 100 μL of IS (10 μg/mL), and (C) subject’s plasma samples, which was spiked with 100 μL of IS (10 μg/mL), of 3 h after oral administration of 2 tablets of 1 mg-azelastine. No significant interference of analytes around retention time of azelastine (0.92 min) or IS (1.16 min) was observed in the mass chromatogram of human blank plasma under LC-ESI/MS/MS conditions. In general, the combination of isocratic HPLC with ESI/MS/MS system leads to short retention time and yields both high selectivity and sensitivity (21).

The analytical calibration curves were constructed with six non-zero standards (10, 50, 100, 500, 1000 and 5000 pg/mL) ranging from 10 to 5000 pg/mL. The calibration curves showed good linearity within the range of 10 (LLOQ) to 5000 pg/mL (Y=0.000045·X + 0.000031, r^2^=0.9930, n=7; Y=peak area ratio, X=plasma concentration of azelastine). As presented in Table 1, the method allowed good precision and accuracy. The inter- and intra-day precisions (%CV) of 30 (LOQ), 500 and 4000 pg/mL were 4.13~8.15 and 11.06~13.86%, respectively. The inter- and intra-day accuracies were 87.57~109.70% and 105.05~108.50%, respectively. The acceptance criteria for precision and accuracy deviation values satisfied with the FDA criteria which are within ±15% of actual values. Under the described analytical conditions, LLOQ (10 pg/mL) defined as the lower concentration at which both precision (11.72~17.91%) and accuracy (93.20~109.10%) were less than or equal to 20% (22). The sensitivity of LC-ESI/MS/MS was very high and the LLOQ detection was sufficient for PK or BE study of two brands of 2 mg-azelastine in any clinical situations. Therefore, LC-ESI/MS/MS system should be regarded as an optimum method for analysis of azelastine concentration in clinical plasma samples with high sensitivity, simple sample preparation process, good precision and accuracy.

**Table 1:** The Method Validation for Analysis of Azelastine in Human Plasma

Concentration (pg/mL)	10 (LLOQ)	30 (LOQ)	500	4000

Inter-day Validation				
Batch1	12.23	29.41	433.05	3459.75
Batch2	10.72	34.67	472.42	4079.98
Batch3	10.50	36.51	438.29	3871.62
Batch4	8.83	35.43	471.86	4127.23
Batch5	11.37	35.28	463.40	3874.12
Average	10.73	34.26	455.80	3882.54
SD	1.26	2.79	18.82	263.53
%CV	11.72	8.15	4.13	6.79
Accuracy (%)	93.20	87.57	109.70	103.03
Intra-day Validation				
Batch A	10.34	30.56	430.45	3498.04
Batch B	10.30	30.61	428.79	4211.69
Batch C	7.57	24.24	562.19	3399.41
Batch D	7.18	23.48	532.28	3345.29
Batch E	10.44	29.36	426.03	4092.86
Average	9.17	27.65	475.95	3709.46
SD	1.64	3.51	65.95	410.09
%CV	17.91	12.68	13.86	11.06
Accuracy (%)	109.10	108.50	105.05	107.83

LLOQ, lower limit of quantification; LOQ, low level of quantification; CV, coefficient of variation.

### Clinical Application in Healthy Subjects

**Figure 3:**
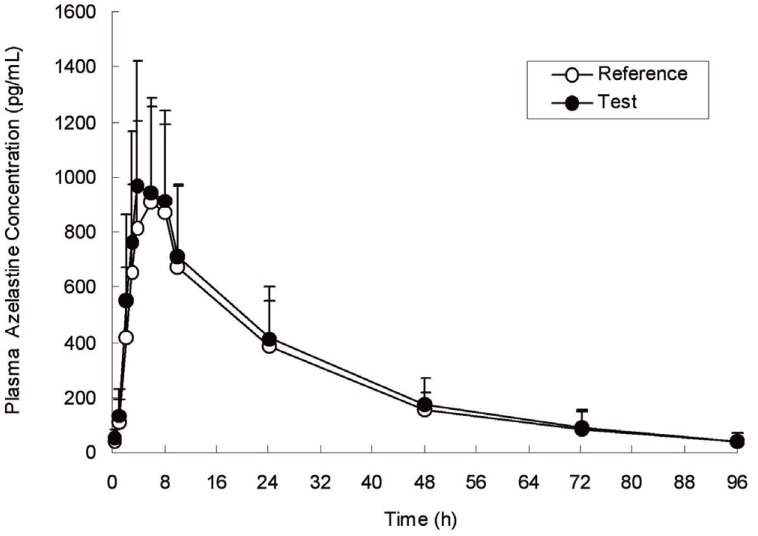
Mean (±S.D.) plasma concentration-time plots of reference and test drugs after an oral administration of 2 tablets of 1 mg-azelastine formulation in 23 healthy Korean male volunteers.

The LC-ESI/MS/MS method was applied to determine azelastine in plasma samples for the purpose of establishing the PK and BE of 2 brands of azelastine formulations in healthy Korean male volunteers. Typical plasma concentration versus time profiles is presented in Figure 3. The closely similar patterns were exhibited through the profiles of the plasma azelastine concentration versus time during 0~96 h after an oral dose of both brands of 2 tablets of 1 mg-azelastine formulations in 23 subjects. The average plasma concentrations of azelastine were in the standard curve range and remained above the BQL (below quantification limit, 10 pg/mL) for the entire sampling period. The basic PK parameters for the reference and test drugs were described in Table 2. The mean AUC_0-t_ and AUC_0-infinity_ for the reference and test drugs were 24.70 and 26.85 ng·h/mL, and 25.96 and 28.24 ng·h/mL, respectively. The mean C_max_ and T_max_ (range), which is independent to sampling time, for the reference and test drugs were 1.02 and 1.10 ng/mL, and 5.9 (4.0~6.0) and 5.6 (4.0~10.0) h, respectively. The mean T_1/2_ and *Ke* for the reference and test drugs were 20.4 and 21.4 h, and 0.0339 and 0.0324 (h^-1^), respectively. No differences between the reference and test formulations were detected at p<0.05 in all the tested parameters. The present C_max_, T_max_ and *Ke* were similar to the previous data for 72 h after an oral dose of 2.2 mg-azelastine in 12 healthy young subjects by RIA method, while AUC_0-infinity_ and T_1/2_ were lower than those data (8). The present PK results showed similar T_max_, *Ke* and T_1/2_ when compared to our previous preliminary results for 96 h after an oral dose of 2 mg-azelastine in 18 healthy young Korean volunteers (23), however AUC_0-t_ and AUC_0-infinity_ were somewhat higher this time. Other results after an oral dose over 2 mg-azelastine in healthy young volunteers showed also similar T_max_, T_1/2_ and *Ke*, and they presented higher AUC_0-t_, AUC_0-infinity_ and C_max_ which were dose-dependent PK parameters (8, 7). When compared to the results from the intra-nasally administered 1.04 mg-azelastine in 29 healthy subjects (24), T_max_ (2~3 h) was shorter than the present data (5.6~5.9 h), resulting in very speedy absorption into the bloodstream, even though C_max_ was similar to the present data. That could be a possible reason that the oral dosage of azelastine is usually prescribed to all the patients with ‘prophylactic and maintenance therapy of bronchilar asthma’ or ‘prophylaxis and treatment of allergic rhinitis’, while the nasal spray dosage of azelastine is more applicable to the patients with acute seasonal allergic rhinitis (SAR) or nonallergic vasomotor rhinitis (VMR) in particular (25).

**Table 2:** The Pharmacokinetic Parameters (mean±SD) After an Oral Dosage of 2 Tablets of 1 mg-Azelastine of Reference and Test Formulations in 23 healthy Korean male volunteers

Parameters	Reference	Test

AUC_0-t_(ng·h/mL)	24.70 ± 10.11	26.85 ± 10.48
AUC_0-infinity_(ng·h/mL)	25.96 ± 10.84	28.24 ± 11.09
Extrapolation(%)	4.8 ± 3.5	4.8 ± 3.6
C_max_(ng/mL)	1.02 ± 0.37	1.10 ± 0.43
T_max_(h)	5.9 ± 1.7 (4.0~6.0)	5.6 ± 1.7 (4.0~10.0)
T_1/2_(h)	20.4 ± 6.0	21.4 ± 6.5
*Ke*(h^-1^)	0.0339 ± 0.0038	0.0324 ± 0.0041

AUC, area under the curve; Extrapolation, (AUC_t-infinity_ / AUC_0-infinity_) × 100; C_max_, peak plasma concentration; T_max_, time for the peak plasma concentration; T_1/2_, half-life; *Ke*, elimination rate constant.

For the BE assessment, Table 3 showed the 90% confidence interval (CI) obtained by analysis of variance (ANOVA) for these parameters after log-transformation of the data. The geometric means and 90% CI of test/reference ratios were 1.095 (0.9769~1.2281) for AUC_0-t_, 1.095 (0.9821~1.2212) for AUC_0-infinity_, and 1.083 (0.9602~1.2233) for C_max_, respectively. Each data was resided in the BE limits (0.8~1.25) and no differences between the reference and test formulations were detected (17, 26).

**Table 3:** The Statistical Evaluation of Bioequivalence of Pharmacokinetic Parameters After an Oral Dosage of 2 Tablets of 1 mg-Azelastine of Reference and Test Formulations in 23 healthy Korean male volunteers

	AUC_0-t_	AUC_0-infinity_	C_max_

Point estimate	1.095	1.095	1.083
90 % CI	0.9769~1.2281	0.9821~1.2212	0.9602~1.2233

The data were presented as ratio of geometric means and the ranges of each PK parameter. CI, Confidence interval.

## CONCLUSION

The LC-ESI/MS/MS method consisted of a simple and sensitive liquid-liquid extraction procedure that successfully applied to PK and BE study of azelastine formulations in 23 healthy Korean male volunteers. The reference and test drugs of 2 tablets of 1 mg-azelastine formulation could be considered bioequivalent based on the obtained its plasma concentrations and corresponding PK parameters. Therefore, the LC-ESI/MS/MS method is readily applicable to routine PK and BE studies of azelastine compounds in the therapeutic drug monitoring and various clinical settings.
